# Supplementary material for: Modifiable lifestyle factors and the risk of post-COVID-19 multisystem sequelae, hospitalization, and death
Source: Nat Commun. 2024 Jul 29;15:6363. doi: 10.1038/s41467-024-50495-7 (PMC11286928; doi:10.1038/s41467-024-50495-7)
Supplement: Supplementary file 1 — Supplementary Information [file 41467_2024_50495_MOESM1_ESM.pdf]

Modifiable lifestyle factors and the risk of post-COVID-19 multisystem sequelae, hospitalization, and death

Yunhe Wang, Binbin Su, Marta Alcalde-Herraiz, Nicola L. Barclay, Yaohua Tian, Chunxiao Li, Nicholas J. Wareham, Roger Paredes, Junqing Xie, Daniel Prieto-Alhambra

Nature Communications, 2024

SUPPLEMENTARY INFORMATION

Table of Contents

Supplementary Note 2
Supplementary Methods 3
Supplementary Tables 4
Supplementary Table 1. Detailed definitions on measurement and classification of lifestyle factors 4
Supplementary Table 2. Distributions of lifestyle score and categories 6
Supplementary Table 3. Definitions and weights of multisystem sequelae 7
Supplementary Table 4. Number of participants with medical conditions between baseline and infection for mediation analyses 10
Supplementary Table 5. Sensitivity analyses of the risk of composite multisystem sequelae, death, and hospitalization 11
Supplementary Table 6. Comparison of the association between healthy lifestyle and composite multisystem sequelae, death, and hospitalization among participants with SARS-CoV-2 infection to those without infection 12
Supplementary Fig. 1. Study design, cohort construction, and timeline 13
Supplementary Fig. 2. Change in lifestyle factors between baseline and the latest repeat assessment for those who undertook both visits 14

Supplementary Note

Evidence before this study

We searched PubMed and MEDLINE for articles published between March 1, 2020, and December 1, 2023, using the search terms “healthy lifestyle”, “risk factor”, “post-COVID condition”, “long COVID”, “post-acute sequelae”, “prevention”, “management”, and “treatment”, with no language restrictions. Previous evidence on the prevention and management of long COVID has mainly focused on vaccination and pharmaceutical approaches, including antivirals (e.g., molnupiravir and nirmatrelvir) and other drugs (e.g., metformin). Vaccination before infection or use of antivirals in selected high-risk patients during acute infection only partially mediates the risk of COVID-19 sequelae. Evidence for the non-pharmaceutical prevention strategies are lacking. We identified only two publications on the association between healthy lifestyle and post-COVID condition, and one meta-analysis of the risk factors for long COVID symptoms. A cross-sectional study of 1981 women suggested an inverse association between healthy lifestyle factors and self-reported symptoms following infection of non-Omicron variants, which was mainly driven by BMI and sleep duration. Another study suggested an inverse prospective association between healthy lifestyle prior to infection and post-COVID cardiovascular events. High BMI and smoking are risk factors for long COVID mainly in hospitalized patients. We did not find any study that assessed the association between a composite healthy lifestyle and subsequent post-COVID complications or sequelae across organ systems, hospitalization, and death.

Added value of this study

In a prospective, population-based cohort of 68,896 participants with COVID-19, adherence to a healthy lifestyle prior to infection was associated with a substantially lower risk of multisystem sequelae (by 20%-36%), death (by 26%-41%), and hospital admission (by 13%-22%) following COVID-19. The reduced risk of sequelae was evident across 10 prespecified organ systems, including cardiovascular, coagulation and hematologic, metabolic and endocrine, gastrointestinal, kidney, mental health, musculoskeletal, neurologic, and respiratory disorders, as well as general symptoms of fatigue and malaise. The reduced risk of multisystem sequelae, hospitalization, and death associated with a healthy lifestyle was consistently observed across participants, regardless of their vaccination status, disease severity, and major SARS-CoV-2 variants, and largely independent of relevant comorbidities. Adherence to a healthy lifestyle prior to infection was consistently and directly associated with reduced risk of sequelae and other adverse health outcomes following COVID-19.

Implications of all the available evidence

The inverse association of healthy lifestyle with multisystem sequelae was even larger than those observed in previous studies of pharmaceutical interventions in non-hospitalized patients. Considering the restricted scope of currently available therapies, such as antivirals (only selected patients at higher risk are qualified during the acute infection) and limited efficacy of vaccination in preventing long COVID, adherence to a healthy lifestyle, in combination with vaccination and, if necessary, potential medications, emerges as practical prevention and care strategies to mitigate the long-term health consequences of SARS-CoV-2 infection. These strategies are of significant clinical and public health importance in reducing the overall burden of post-COVID conditions and improving preparedness for future pandemics.

Supplementary Methods

Causal mediation analysis

We used causal mediation analysis to specifically evaluate the extent to which a habitual healthy lifestyle may affect an COVID-19 sequelae outcome through a potential pathway of relevant medical conditions (mediator) prior to the infection. The mediator of interest was defined as relevant events that occurred between the baseline lifestyle assessment and the date of COVID-19 diagnosis. The statistical analysis was executed in a four-step process: (1) we first fitted a logistic regression model with the mediator as dependent variables and all covariates from the primary analysis as independent variables. (2) another logistic regression model was then fitted with multisystem sequelae as the dependent variable and the corresponding mediator along with other covariates as independent variables. (3) the direct effects (DE), indirect effects and their 95% confidence intervals (IE) were estimated using quasi-Bayesian Monte Carlo methods with 1,000 simulations for each. (4) finally, the mediation proportion was computed as IE/(IE+DE)*100.
Directed acyclic graph (DAG) for identifying confounding variables:

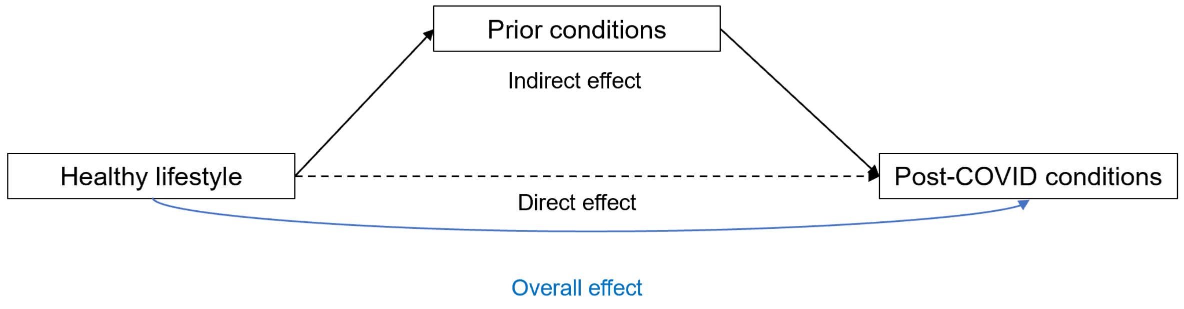

Quantitative sensitivity analysis

Quantitative sensitivity analysis was used to adjust for changes in lifestyle factors over time since the baseline assessment. We used a standard algebraic approach that recalculates the expected cell frequencies for a correctly classified 2 x 2 contingency table based on the observed frequencies in a misclassified table. We then derived the bias-adjusted odds ratio using the formula A*D/B*C, assuming non-differential sensitivity and specificity both at 0.9. The equations used to calculate the expected true data, considering exposure misclassification, are as follows:

	**Observed**		**Misclassification adjusted data**	
	***E*1**	***E*0**	***E*1**	***E*0**
*D*1	*a*	*b*	[*a* − *D*1 Total (1 − SP*D*1)]/(SE*D*1–1 + SP*D*1)	*D*1 Total − *A*
*D*0	*c*	*d*	[*c* − *D*0 Total (1 − SP*D*0)]/(SE*D*0–1 + SP*D*0)	*D*0 Total − *C*
Total	*a* + *c*	*b* + *d*	*A* + *C*	*B* + *D*

Here, SED1 and SED0 denote the sensitivity in cases and non-cases, respectively, while SPD1 and SPD0 represent specificity in cases and non-cases, respectively.

Supplementary Tables

Supplementary Table 1. Detailed definitions on measurement and classification of lifestyle factors

Modifiable lifestyle factor	Source and guidelines	UK Biobank questionnaire/definitions	Binary category	
			Healthy (1 point)	Unhealthy (0 point)
**Smoking status**	UK national health guidelines recommend smoking cessation for better health: such as National Health Service (NHS) *Live Well* guideline: Quit smoking and guidance QS82 from NICE: Reducing and preventing tobacco use	"Do you smoke tobacco now?" and "In the past, how often have you smoked tobacco?"	Past or never smoker	Current smoker
**Alcohol consumption**	NHS *Live Well* guideline: alcohol advice recommends to drink no more than 14 units of alcohol a week, spread across 3 days or more and there's no completely safe level of drinking. The US 2020-2025 Dietary Guidelines for Americans suggest that adults of legal drinking age can choose not to drink or to drink in moderation by limiting intake to 2 drinks or less in a day for men and 1 drink or less in a day for women when alcohol is consumed.	"About how often do you drink alcohol?"	≤ 4 times week	Daily or almost daily
**BMI**	NHS *Live Well* guideline and a recent meta-analysis on risk factors for long COVID suggest that obesity is a serious health concern that increases the risk of many health outcomes, including post-COVID conditions.	Height was measured in whole centimeters with a Seca 202 device. Weight was measured to the nearest 0.1 kg. BMI was calculated as weight in kilograms divided by the square of height in meters.	BMI <30 kg/m^2	BMI ≥ 30 kg/m^2
**Physical activity**	WHO guidelines on physical activity and NHS *Live Well* guideline suggest moderate- and vigorous-intensity physical activity improve health. All adults should undertake 150-300 min of moderate-intensity, or 75-150 min of vigorous-intensity physical activity, or some equivalent combination of moderate-intensity and vigorous-intensity aerobic physical activity, per week.	IPAQ short form 2[a]– total time walking or moderate and vigorous-intensity PA in previous week	≥150 min/week moderate or ≥ 75 min/week vigorous PA	< 75 min/week vigorous PA
**TV viewing/sedentary time**	WHO guidelines on sedentary behaviour and NHS *Live Well* guideline: reducing sedentary behaviors is recommended across all age groups and abilities	"In a typical day, how many hours do you spend watching TV?"[b]	< 4 h/day	≥ 4 h/day
**Sleep duration**	In accordance with NHS *Live Well* guideline, American Academy of Sleep Medicine (AASM) and Sleep Research Society (SRS) recommendation, sleep duration of 7 to 9	"About how many hours sleep do you get in every 24 hours?"	7-9 h/day	<7 or >9h/day

	hours was classified into healthy lifestyle category.			
**Fruit and vegetable intake**	Consuming a healthy diet throughout the life-course helps to prevent malnutrition in all its forms as well as a range of noncommunicable diseases and conditions. NHS *Eat Well* guideline, WHO guideline on healthy diet, and World Cancer Research Fund International suggest that at least 400 g, or five portions, of fruit and vegetables per day; at least 2 portions of fish a week, including 1 of oily fish; limit consumption to red meat and processed meat (though threshold varied by guidelines: NHS suggests keep intake of red and processed meat to 70g or less a day; WCRF suggest limit consumption to no more than about three portions per week (equivalent to about 350–500g (about 12–18oz) cooked weight.) and consume very little, if any, processed meat).	"About how many of …. would you eat per day?" Separate questions for pieces of fresh and dried fruit, tablespoons of salad or cooked/raw vegetables. Combined and converted to g/day (1 portion = 80 g)	≥ 400 g/day	<400 g/day
**Oily fish intake**		"How often do you eat oily fish? (e.g. sardines, salmon, mackerel, herring)"	≥1 portion/week	<1 portion/week
**Red meat intake**		"How often do you eat…?" Separate questions for Beef / lamb or mutton / pork (excluding processed meats such as ham or bacon). Red meat included due to clear link between red meat and mortality.	≤3 portion/week	>3 portion/week
**Processed meat intake**		"How often do you eat processed meats (such as bacon, ham, sausages, meat pies, kebabs, burgers, chicken nuggets)?"	≤1 portion/week	>1 portion/week

[a]UK Biobank physical activity (PA) data were analyzed in accordance with the International Physical Activity Questionnaire (IPAQ) scoring protocol, with total physical activity computed as the sum of walking, moderate, and vigorous activity, measured as metabolic equivalents (MET-hours/week).
[b]UK Biobank participants were asked about work-related sitting time and were not asked about discretionary/leisure time sedentary behavior. Therefore, TV viewing time, which is strongly associated with health outcomes, was used instead of sedentary time. UK Biobank participants were asked separately about time spent using a computer, excluding computer time at work. This information was used to check for implausible values for TV viewing time.

Supplementary Table 2. Distributions of lifestyle score and categories

A, All eligible participants

Lifestyle category[a]	Lifestyle score	No. of participants (N=472,977)		Percentage, %	
**Unfavorable**	0	6	55400	0.00%	11.7%
	1	97		0.02%	
	2	777		0.16%	
	3	3949		0.83%	
	4	13914		2.94%	
	5	36657		7.75%	
**Intermediate**	6	75141	192407	15.89%	40.7%
	7	117266		24.79%	
**Favorable**	8	127905	225170	27.04%	47.6%
	9	80470		17.01%	
	10	16795		3.55%	

B, COVID-19 cohort

Lifestyle category[a]	Lifestyle score	No. of participants (N=68,896)		Percentage, %	
**Unfavorable**	0	1	8476	0.00%	12.3%
	1	18		0.03%	
	2	122		0.18%	
	3	639		0.93%	
	4	2142		3.11%	
	5	5554		8.06%	
**Intermediate**	6	11258	28457	16.34%	41.3%
	7	17199		24.96%	
**Favorable**	8	18182	31963	26.39%	46.4%
	9	11273		16.36%	
	10	2508		3.64%	

[a]A binary variable was created for each of the 10 lifestyle factors, with 1 point assigned for healthy category and 0 otherwise. A composite lifestyle score was then calculated for each participant by summing the total number of unhealthy lifestyle factors, with a range of 0 to 10. Based on the composite score, participants were classified into three lifestyle categories: unfavorable (0-5), intermediate (6-7), and favorable (8-10).

Supplementary Table 3. Definitions and weights of multisystem sequelae

Organ system	Sequela	Cause of death or disability	ICD-10 code	Weight
Cardiovascular	Acute coronary disease	Ischemic heart disease	I24	1.009
Cardiovascular	Myocardial infarction	Ischemic heart disease	I21,I22	1.009
Cardiovascular	Atrial fibrillation	Atrial fibrillation and flutter	I480,I481,I482	0.124
Cardiovascular	Atrial flutter	Atrial fibrillation and flutter	I483,I484	0.124
Cardiovascular	Angina	Chest pain	I20	0.138
Cardiovascular	Bradycardia	Atrial fibrillation and flutter	R001	0.124
Cardiovascular	Cardiac arrest	Ischemic heart disease	I46	1.009
Cardiovascular	Non-ischemic cardiomyopathy	Other cardiomyopathy	I42,I43,B332	0.740
Cardiovascular	Cariogenic shock	Ischemic heart disease	R570	1.009
Cardiovascular	Heart failure	Ischemic heart disease	I50	1.009
Cardiovascular	Ischemic cardiomyopathy	Other cardiomyopathy	I255	0.740
Cardiovascular	Myocarditis	Myocarditis	I514	0.584
Cardiovascular	Pericarditis	Myocarditis	I30,I311,I312,I313,I318,I319,I32	0.584
Cardiovascular	Tachycardia	Atrial fibrillation and flutter	R000	0.124
Cardiovascular	Ventricular arrhythmias	Atrial fibrillation and flutter	I490,I470,I471,I472	0.124
Coagulation	Anemia	Anemia	D60,D61,D63,D64,D62	0.002
Coagulation	Deep vein thrombosis	Stroke	I824,I825	0.540
Coagulation	Coagulation defect	Stroke	D689	0.540
Coagulation	Pulmonary embolism	Stroke	I26	0.540
Coagulation	Venous thomobilic embolism	Stroke	I820,I821,I822,I823,I826,I827,I82A,I82B,I82C,I828,I829	0.540
Diabetes	Diabetes	Diabetes mellitus type 2	E10,E11	0.114
Gastrointestinal	Abdominal pain	Abdominal pain	R100,R101,R103,R108,R109	0.124
Gastrointestinal	Acute gastritis	Gastritis and duodenitis	K290	0.047
Gastrointestinal	Acute pancreatitis	Pancreatitis	K85	0.503
Gastrointestinal	Cholangitis	Gallbladder and biliary diseases	K830	0.031
Gastrointestinal	Constipation	Constipation	K590	0.022
Gastrointestinal	Diarrhea	Moderate diarrheal diseases	K591,R197	0.188
Gastrointestinal	Gastroesophageal reflux disease	Gastroesophageal reflux disease	K21	0.008
Gastrointestinal	Irritable bowel syndrome	Inflammatory bowel disease	K55	0.282
Gastrointestinal	Liver diseases	Total burden related to Non-alcoholic fatty liver disease	K70,K71,K73,K74,K7,K721,Z944,B18,I850,I859,I982	0.005
Gastrointestinal	Peptic ulcer disease	Peptic ulcer disease	K25,K26,K27,K28	0.214
Gastrointestinal	Vomiting	Vomiting	R111	0.223
General	Fatigue and malaise	Fatigue and malaise*	R53	0.215
Kidney	Acute kidney injury	Acute kidney disease	N17,N19	0.605

Kidney	Chronic Kidney Disease	Chronic kidney disease	N18	0.057
Mental health	Psychotic disorders	Schizophrenia	F20,F21,F22,F23,F24,F25,F26,F27,F28,F29	0.049
Mental health	Mania/Bipolar affective disorder	Depressive disorders	F30,F31	0.136
Mental health	Depressive episode	Depressive disorders	F32	0.136
Mental health	Phobic anxiety disorder	Anxiety disorders	F40	0.093
Mental health	Panic disorder	Anxiety disorders	F410	0.093
Mental health	Generalized anxiety disorder	Anxiety disorders	F411	0.093
Mental health	Posttraumatic stress disorder	Anxiety disorders	F431	0.093
Mental health	Substance use disorders	Drug use disorders	F10,F11,F12,F13,F14,F15,F16,F17,F18,F19	0.621
Mental health	Sleep disorders	Fatigue*	F51,G47	0.215
Musculoskeletal	Joint pain	Musculoskeletal disorders	M255	0.110
Musculoskeletal	Myalgias	Musculoskeletal disorders	M791	0.110
Musculoskeletal	Myopathy	Musculoskeletal disorders	G72	0.110
Musculoskeletal	Osteoarthritis	Musculoskeletal disorders	M15,M16,M17,M18,M19	0.110
Neurologic	Abnormal involuntary movements	Parkinson's disease	R25	0.796
Neurologic	Alzheimer’s disease	Alzheimer's disease and other dementias	G30	0.413
Neurologic	Memory problems	Alzheimer's disease and other dementias	R413	0.413
Neurologic	Central Venous thrombosis	Stroke	I636	0.540
Neurologic	Dizziness	Fatigue*	R42	0.215
Neurologic	Dysautonomia	Neuropathy	G900,G903,G904,G905,G908,G909	0.133
Neurologic	Dystonia	Parkinson's disease	G24	0.796
Neurologic	Bells palsy	Neuropathy	G510	0.133
Neurologic	Headache disorders	Tension-type headache	G44	0.002
Neurologic	Loss of hearing	Age-related and other hearing loss	H90,H91,H93	0.031
Neurologic	Migraine	Migraine	G43	0.037
Neurologic	Neurocognitive decline	Cognitive symptoms*	G454,F440,F04, F01,F02,G311,G318,G319	0.128
Neurologic	Paresthesia	Neuropathy	R202	0.133
Neurologic	Parkinsons-like disease	Parkinson's disease	G20	0.796
Neurologic	Polyneuropathy	Neuropathy	G611,G618,G619,G62	0.133
Neurologic	Epilepsy and seizures	Idiopathic epilepsy	G40	0.303
Neurologic	Loss of smell	Other sense organ diseases	R430,R431	0.023
Neurologic	Somnolence	Fatigue*	R400	0.215
Neurologic	Ischemic stroke	Stroke	G46,I63	0.540
Neurologic	Loss of taste	Other sense organ diseases	R439	0.023
Neurologic	Transient cerebral ischemic attack	Transient cerebral ischemic attack	G45	0.250
Neurologic	Tremor	Parkinson's disease	R251	0.796

Neurologic	Vision abnormalities	Vision problems	H53,H54,H34,H356	0.036
Pulmonary	Cough	Mild and moderate respiratory disease*	R05	0.138
Pulmonary	Hypoxemia	Severe respiratory disease*	R090	0.410
Pulmonary	Interstitial lung disease	Interstitial lung disease	J841, J848, J849	0.112
Pulmonary	Shortness of breath	Mild and moderate respiratory disease*	R060	0.138

Health burden coefficients were obtained from T. Vos et al., Global burden of 369 diseases and injuries in 204 countries and territories, 1990–2019: a systematic analysis for the Global Burden of Disease Study 2019. The Lancet 396, 1204-1222 (2020) and other GBD sources: *Health burden weights were obtained from S. W. Hanson et al., Estimated global proportions of individuals with persistent fatigue, cognitive, and respiratory symptom clusters following symptomatic COVID-19 in 2020 and 2021. Jama 328, 1604-1615 (2022). In sensitivity analysis, the weighted score was calculated for each participant by summing the weights of all incident sequelae during the follow-up period.

The selection and definition of prespecified multisystem sequelae of COVID-19 were based on literature review and previous knowledge:

High-dimensional characterization of post-acute sequelae of COVID-19. Nature
Risk of clinical sequelae after the acute phase of SARS-CoV-2 infection: retrospective cohort study. BMJ
Long-term cardiovascular outcomes of COVID-19. Nat Med
Risks and burdens of incident diabetes in long COVID: a cohort study. Lancet Diabetes Endocrinol
Risks and burdens of incident dyslipidaemia in long COVID: a cohort study. Lancet Diabetes Endocrinol
Long-term neurologic outcomes of COVID-19. Nat Med
Risks of mental health outcomes in people with COVID-19. BMJ
Kidney outcomes in long COVID. J. Am. Soc. Nephrol.
Long-term gastrointestinal outcomes of COVID-19. Nat Commun
Postacute sequelae of COVID-19 at 2 years. Nat Med

Supplementary Table 4. Number of participants with medical conditions between baseline and infection for mediation analyses

Pre-infection medical conditions[a]	Participants (%)
Any complication	42.86
General fatigue	1.29
Coagulation diseases	5.01
Neurologic diseases	11.57
Pulmonary diseases	3.56
Kidney diseases	5.64
Gastrointestinal diseases	17.51
Mental disorders	12.25
Musculoskeletal diseases	14.03
Cardiovascular diseases	10.44
Diabetes	7.54

[a]Defined using the same ICD codes as post-infection sequelae in Supplementary Table 3.

Supplementary Table 5. Sensitivity analyses of the risk of composite multisystem sequelae, death, and hospitalization

A,

	**Multisystem sequelae (HR and 95% CI)**		**Death (HR and 95% CI)**		**Hospitalization (HR and 95% CI)**	
	Intermediate	Favorable	Intermediate	Favorable	Intermediate	Favorable
**Assigning weight to each individual sequela and using zero inflated Poisson regression**	0.78 (0.70-0.88)	0.56 (0.50-0.64)	NA	NA	NA	NA
**Extending the washout period from one year to two years**	0.80 (0.73-0.88)	0.65 (0.59-0.72)	0.74 (0.66-0.84)	0.59 (0.52-0.66)	0.90 (0.83-0.98)	0.83 (0.76-0.90)
**Defining events of post-acute sequelae 90 days after infection**	0.83 (0.73-0.95)	0.71 (0.62-0.81)	0.66 (0.49-0.88)	0.57 (0.42-0.77)	0.95 (0.86-1.03)	0.88 (0.81-0.97)
**Using only the first three ICD disease diagnoses to identify outcomes**	0.73 (0.62-0.86)	0.64 (0.54-0.76)	0.81 (0.70-0.94)	0.59 (0.50-0.69)	0.75 (0.67-0.85)	0.59 (0.52-0.67)
**Excluding BMI from composite lifestyle index**	0.84 (0.76-0.93)	0.77 (0.70-0.85)	0.77 (0.67-0.89)	0.64 (0.56-0.74)	0.93 (0.85-1.02)	0.90 (0.82-0.98)

B, account for potential misclassification of lifestyle factors[a]

	**Multisystem sequelae (OR and 95% CI)**		**Death (OR and 95% CI)**		**Hospitalization (OR and 95% CI)**	
	Intermediate	Favorable	Intermediate	Favorable	Intermediate	Favorable
**Without adjustment**	0.75 (0.69-0.81)	0.54 (0.50-0.59)	0.74 (0.69-0.79)	0.50 (0.47-0.54)	0.74 (0.67-0.81)	0.53 (0.48-0.58)
**Adjusted for potential misclassification**	0.70 (0.63-0.77)	0.47 (0.42-0.52)	0.69 (0.63-0.74)	0.43 (0.39-0.47)	0.68 (0.61-0.77)	0.45 (0.40-0.51)

A, qualitative sensitivity analyses. B, quantitative sensitivity analysis accounting for potential misclassification of lifestyle factors over time. [a]Odds ratios were used to quantify associations and assumed a sensitivity and specificity of 90% for each lifestyle component.

Supplementary Table 6. Comparison of the association between healthy lifestyle and composite multisystem sequelae, death, and hospitalization among participants with SARS-CoV-2 infection to those without infection

	Multisystem sequelae (HR and 95% CI)		Death (HR and 95% CI)		Hospitalization (HR and 95% CI)	
	Intermediate	Favorable	Intermediate	Favorable	Intermediate	Favorable
**Overall (0-210 days following infection or index date)**						
With SARS-CoV-2 infection (N=60,565/68,894/55,114)	0.80 (0.74-0.87)	0.64 (0.58-0.69)	0.74 (0.66-0.84)	0.59 (0.52-0.66)	0.87 (0.81-0.93)	0.78 (0.73-0.84)
Without SARS-CoV-2 infection (N = 352,945/395,391/320,642)	0.80 (0.77-0.84)	0.69 (0.66-0.72)	0.69 (0.62-0.77)	0.57 (0.51-0.64)	0.91 (0.87-0.94)	0.85 (0.82-0.88)
**Acute (0-30 days following infection or index date)**						
With SARS-CoV-2 infection	0.77 (0.69-0.87)	0.57 (0.50-0.64)	0.79 (0.68-0.91)	0.61 (0.52-0.72)	0.79 (0.71-0.88)	0.64 (0.57-0.71)
Without SARS-CoV-2 infection	0.90 (0.80-1.00)	0.77 (0.68-0.86)	0.59 (0.44-0.77)	0.50 (0.37-0.66)	0.93 (0.85-1.02)	0.87 (0.79-0.95)
**Post-acute (30-210 days following infection or index date)**						
With SARS-CoV-2 infection	0.83 (0.75-0.92)	0.69 (0.62-0.77)	0.67 (0.55-0.81)	0.54 (0.44-0.66)	0.95 (0.87-1.04)	0.88 (0.81-0.97)
Without SARS-CoV-2 infection	0.79 (0.75-0.82)	0.67 (0.64-0.70)	0.71 (0.63-0.80)	0.59 (0.52-0.67)	0.88 (0.85-0.90)	0.79 (0.77-0.82)

To validate and compare the beneficial effects of healthy lifestyle on predefined PCC outcomes observed in participants with COVID-19 versus those with no evidence of infection. We use a parallel comparison setting where we assess the association between healthy lifestyle and adverse health outcomes in participants with no evidence of infection, and we compared the risk estimates with those derived from the infected group.

Supplementary Fig. 1. Study design, cohort construction, and timeline

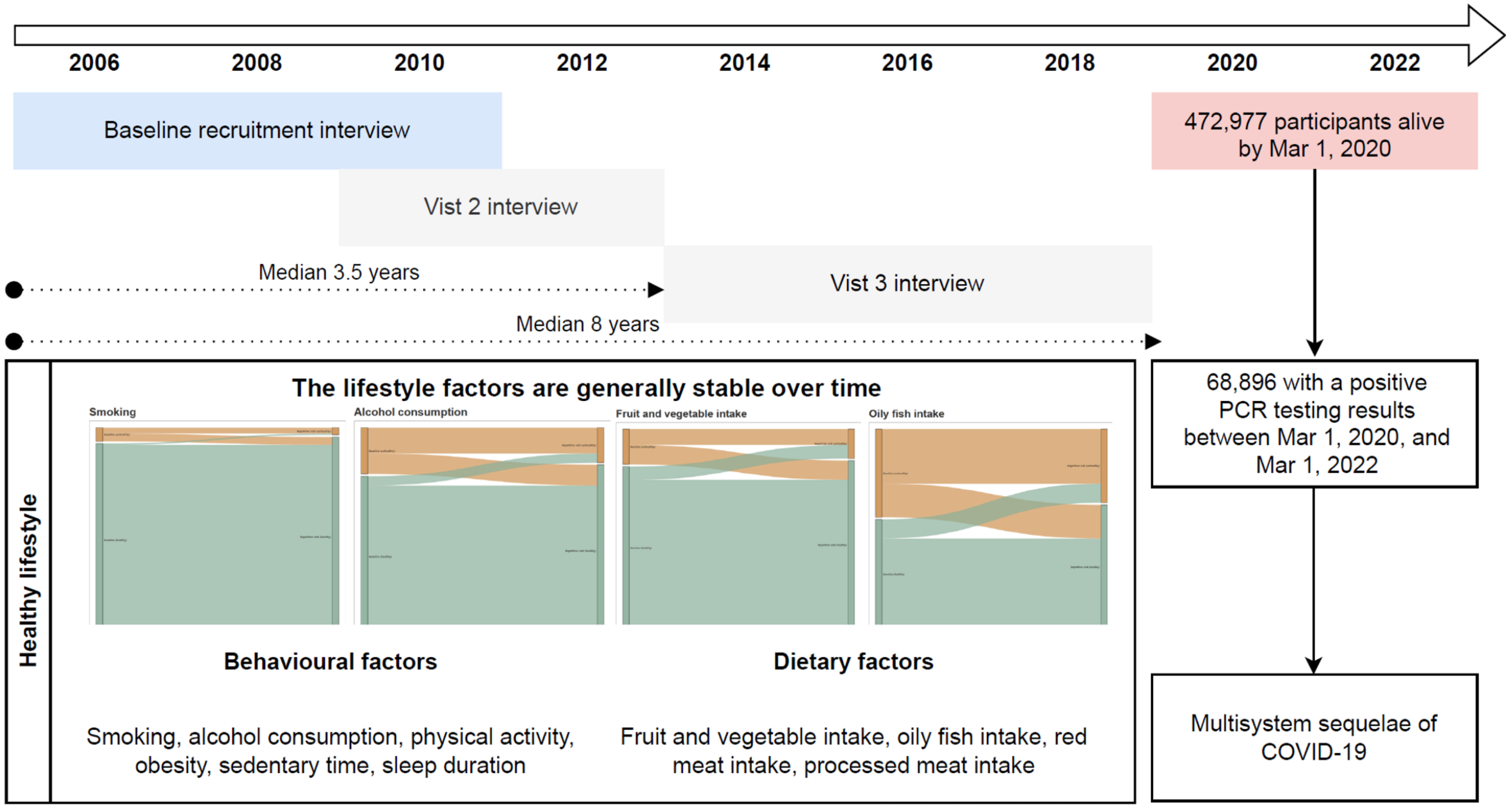

Supplementary Fig. 2. Change in lifestyle factors between baseline and the latest repeat assessment for those who undertook both visits

a, Change in individual lifestyle factor (unhealthy and healthy)

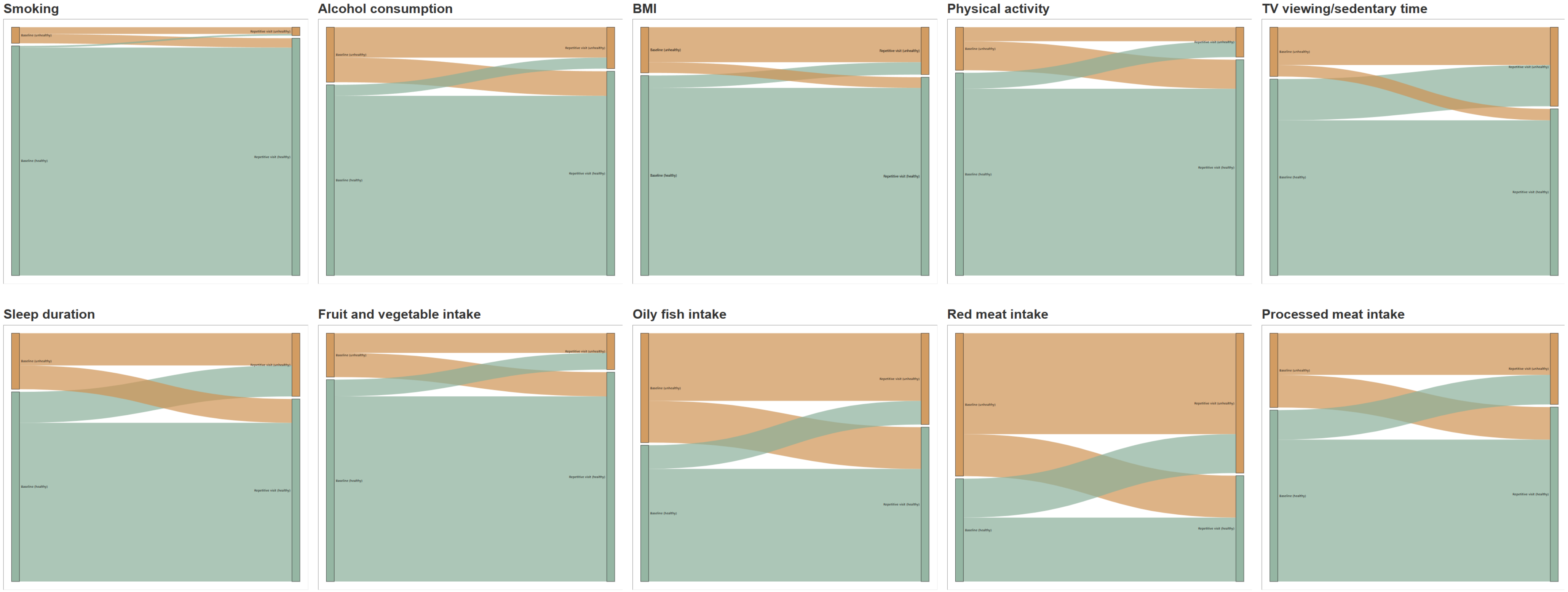

b Change in composite lifestyle category (unfavorable, intermediate, and favorable)

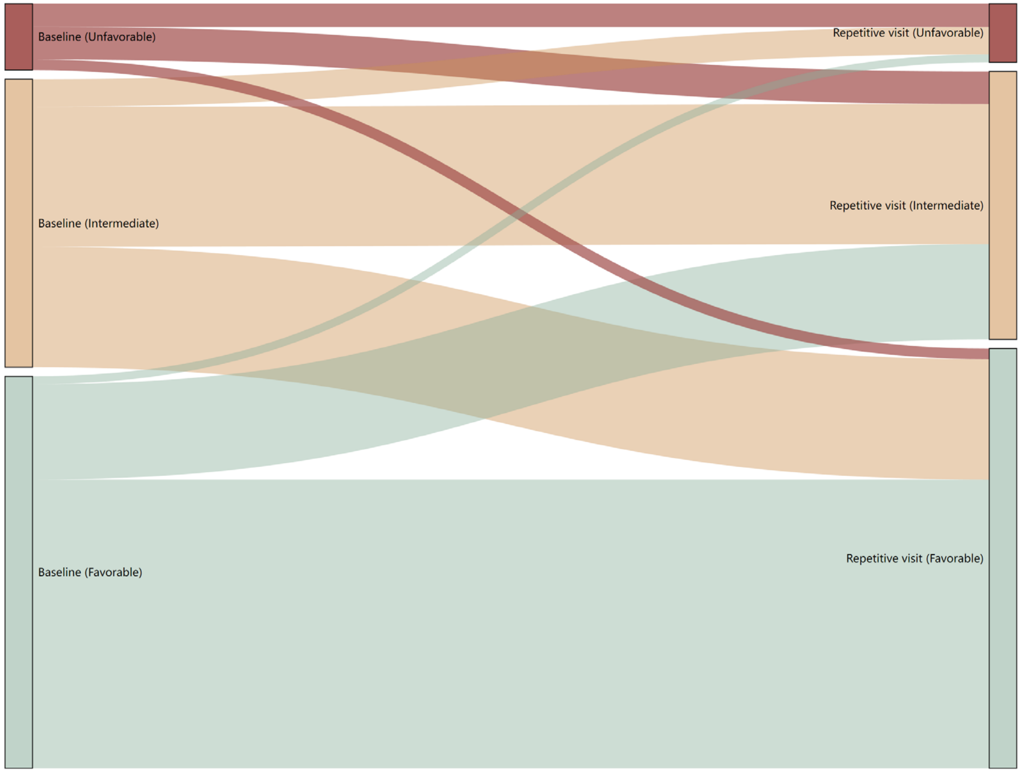

a, Change in 10 individual lifestyle factor between baseline and the latest repeat assessment. **b**, Change in composite lifestyle category between baseline and the latest repeat assessment. Left column of each plot indicates baseline and right column indicates repeat assessment. For each individual lifestyle factor as binary variable, unhealthy category is in orange, favorable category in green. For each the composite lifestyle as categorical variable, unfavorable category is in red, intermediate category in yellow, and favorable category in green. 34.9% of participants with an unfavorable lifestyle, 48.6% with an intermediate lifestyle, and 73.7% with a favorable lifestyle at baseline remained in the same corresponding lifestyle category at the latest repeat assessment ~8 years later (overall proportion of stable categories, 60.6%). The sample size for each factor varied by missing conditions (N=~60,000).
