# Supplementary material for: Modifiable lifestyle factors and the risk of post-COVID-19 multisystem sequelae, hospitalization, and death
Source: Nat Commun. 2024 Jul 29;15:6363. doi: 10.1038/s41467-024-50495-7 (PMC11286928; doi:10.1038/s41467-024-50495-7)
Supplement: Supplementary file 3 — Reporting Summary [file 41467_2024_50495_MOESM3_ESM.pdf]

nature portfolio

Corresponding author(s): Junqing Xie
Last updated by author(s): Jun 22, 2024

Reporting Summary

Nature Portfolio wishes to improve the reproducibility of the work that we publish. This form provides structure for consistency and transparency in reporting. For further information on Nature Portfolio policies, see our Editorial Policies and the Editorial Policy Checklist.

Statistics

For all statistical analyses, confirm that the following items are present in the figure legend, table legend, main text, or Methods section.

n/a	Confirmed	
☐	☒	The exact sample size (*n*) for each experimental group/condition, given as a discrete number and unit of measurement
☒	☐	A statement on whether measurements were taken from distinct samples or whether the same sample was measured repeatedly
☐	☒	The statistical test(s) used AND whether they are one- or two-sided<br>*Only common tests should be described solely by name; describe more complex techniques in the Methods section.*
☐	☒	A description of all covariates tested
☐	☒	A description of any assumptions or corrections, such as tests of normality and adjustment for multiple comparisons
☐	☒	A full description of the statistical parameters including central tendency (e.g. means) or other basic estimates (e.g. regression coefficient) AND variation (e.g. standard deviation) or associated estimates of uncertainty (e.g. confidence intervals)
☐	☒	For null hypothesis testing, the test statistic (e.g. *F*, *t*, *r*) with confidence intervals, effect sizes, degrees of freedom and *P* value noted<br>*Give P values as exact values whenever suitable.*
☒	☐	For Bayesian analysis, information on the choice of priors and Markov chain Monte Carlo settings
☐	☒	For hierarchical and complex designs, identification of the appropriate level for tests and full reporting of outcomes
☒	☐	Estimates of effect sizes (e.g. Cohen's *d*, Pearson's *r*), indicating how they were calculated

Our web collection on statistics for biologists contains articles on many of the points above.

Software and code

Policy information about availability of computer code

Data collection	No software was used.
Data analysis	All analyses and data visualizations were conducted using R statistical software (version 4.1). The code used for this study has been deposited in a public git repository (https://github.com/xjq8065524/).

For manuscripts utilizing custom algorithms or software that are central to the research but not yet described in published literature, software must be made available to editors and reviewers. We strongly encourage code deposition in a community repository (e.g. GitHub). See the Nature Portfolio guidelines for submitting code & software for further information.

Data

Policy information about availability of data

All manuscripts must include a data availability statement. This statement should provide the following information, where applicable:

- Accession codes, unique identifiers, or web links for publicly available datasets
- A description of any restrictions on data availability
- For clinical datasets or third party data, please ensure that the statement adheres to our policy

Researchers can apply to use the UK Biobank dataset by registering and applying at https://ukbiobank.ac.uk/register-apply/. The aggregated data supporting the findings of this study are available within the paper and its supplementary information files. The datasets generated during the current study are not publicly available but can be obtained from the corresponding author, provided that the request aligns with the ethical guidelines and privacy regulations.

Research involving human participants, their data, or biological material

Policy information about studies with human participants or human data. See also policy information about sex, gender (identity/presentation), and sexual orientation and race, ethnicity and racism.

Reporting on sex and gender	Out of 472,977 eligible participants from a large-scale community-based prospective cohort, 68,896 participants with a positive SARS-CoV-2 test result between March 1, 2020 and March 1, 2022 were included in the current study, including 32,098 women and 36,798 men, with a mean [SD] age of 66.6 [8.4] years. All participants provided informed written consent to take part in the study and be followed-up through linkage to health-related records. The main analyses were assessed in subgroups based on sex.
Reporting on race, ethnicity, or other socially relevant groupings	The main analyses were assessed in sex and other population characteristics such as age, ethnicity (White vs other ethnic groups), and virus-related factors (vaccine status, test setting of infection, and variants of SARS-CoV-2).
Population characteristics	Of the COVID-19 cohort, the mean (SD) age was 66.6 (8.4) years, 53.4% were male and 82.1% were White. For composite healthy lifestyle prior to the infection, 12.3% followed an unfavorable lifestyle, 41.3% followed an intermediate lifestyle, and 46.4% followed a favorable lifestyle. The median [IQR] number of healthy lifestyle factors participants engaged in was 7 [6-8]. For prespecified COVID-19 sequalae, 5.5% and 7.8% had sequelae in at least one organ system during the acute and post-acute phase of infection, respectively.
Recruitment	The UK Biobank is an ongoing community-based prospective cohort study, which recruited more than 500,000 participants out of 9.2 million adults aged 40-69 years in the UK who were identified from National Health Service and invited to participants (5.5% response rate). The baseline survey took place from 2006 to 2010 in 22 assessment centers.
Ethics oversight	This study was based on data from UK Biobank. All participants provided written informed consent at the UK Biobank cohort recruitment. This study received ethical approval from UK Biobank Ethics Advisory Committee (EAC) and was performed under the application of 65397.

Note that full information on the approval of the study protocol must also be provided in the manuscript.

Field-specific reporting

Please select the one below that is the best fit for your research. If you are not sure, read the appropriate sections before making your selection.

☒ Life sciences ☐ Behavioural & social sciences ☐ Ecological, evolutionary & environmental sciences

For a reference copy of the document with all sections, see nature.com/documents/nr-reporting-summary-flat.pdf

Life sciences study design

All studies must disclose on these points even when the disclosure is negative.

Sample size	Out of 472,977 eligible UK Biobank participants, 68,896 participants with a positive SARS-CoV-2 test result between March 1, 2020 and March 1, 2022 were included in the current study. To our knowledge, the UK Biobank including about half a million participants is one of the current largest random well-controlled population-based cohort with detailed and robust recording of confounding factors that were largely unavailable in previous studies based on electronic health records. The large sample size obtained were deemed to provide reliable risk estimates of multiple post-COVID-19 multisystem complications and other adverse outcomes.
Data exclusions	Participants with missing data on study exposures and covariates of interest at baseline were further excluded. To avoid potential reverse causality, incident outcomes were assessed in participants with no history of the related outcome within one year before the date of the first infection.
Replication	We conduct repeated the main analysis in uninfected group to compare the effects of healthy lifestyle on adverse outcomes following COVID-19 with the effects among participants without infection. The associations between healthy lifestyle and risk of three predefined main outcomes were largely similar among participants with SARS-CoV-2 infection and those with no evidence of infection during the overall or 30-210 days of follow-up (post-acute phase).
Randomization	No randomization was required as all samples were included in the analysis.
Blinding	No blinding was applicable to this observational study as no intervention were applied to participants.

Reporting for specific materials, systems and methods

We require information from authors about some types of materials, experimental systems and methods used in many studies. Here, indicate whether each material, system or method listed is relevant to your study. If you are not sure if a list item applies to your research, read the appropriate section before selecting a response.

Materials & experimental systems

n/a	Involved in the study	
☒	☐	Antibodies
☒	☐	Eukaryotic cell lines
☒	☐	Palaeontology and archaeology
☒	☐	Animals and other organisms
☒	☐	Clinical data
☒	☐	Dual use research of concern
☒	☐	Plants

Methods

n/a	Involved in the study	
☒	☐	ChIP-seq
☒	☐	Flow cytometry
☒	☐	MRI-based neuroimaging

Plants

Seed stocks	NA
Novel plant genotypes	NA
Authentication	NA
